# Improved vascular health linked to increased physical activity levels and reduced sedentary behavior in rheumatoid arthritis

**DOI:** 10.1152/ajpheart.00640.2024

**Published:** 2024-11-01

**Authors:** K. Meireles, T. Peçanha, A. J. Pinto, L. P. Santos, B. C. Mazzolani, F. I. Smaira, D. Rezende, A. C. M. Ribeiro, A. L. de Sá Pinto, F. R. Lima, N. D. da Silva Junior, C. L. M. Forjaz, B. Gualano, H. Roschel

**Affiliations:** ^1^Applied Physiology and Nutrition Research Group—School of Physical Education and Sport and Faculdade de Medicina FMUSP, Universidade de São Paulo, Sao Paulo, Brazil; ^2^Center of Lifestyle Medicine, Faculdade de Medicina FMUSP, Universidade de Sao Paulo, Sao Paulo, Brazil; ^3^Department of Sport and Exercise Sciences, Manchester Metropolitan University Institute of Sport, Manchester Metropolitan University, Manchester, United Kingdom; ^4^Division of Endocrinology, Metabolism, and Diabetes, Anschutz Health and Wellness Center, School of Medicine, University of Colorado Anschutz Medical Campus, Aurora, Colorado, United States; ^5^Rheumatology Division, Faculdade de Medicina FMUSP, Universidade de Sao Paulo, Sao Paulo, Brazil; ^6^Exercise Hemodynamic Laboratory, School of Physical Education and Sport, University of Sao Paulo, Sao Paulo, Brazil

**Keywords:** autoimmune rheumatic diseases, exercise, inflammation, vasculature

## Abstract

Rheumatoid arthritis (RA) is characterized by deteriorated vascular health and increased cardiovascular risk. Physical activity (PA) is recommended for cardiovascular management in RA, but evidence on the associations between objectively measured PA and vascular health markers in RA is limited. In this cross-sectional study, 82 postmenopausal women with RA (62 ± 7 yr) undertook ultrasound assessments of vascular function and structure, including brachial and superficial femoral artery (BA and SFA) flow-mediated dilation; baseline and post-hyperemia peak diameters; and carotid intima-media thickness. Participants also performed a 7-day accelerometer-based assessment of PA and sedentary behavior (SB). Fitted regression models controlled for age, body mass index, and disease activity were conducted to examine associations between vascular and PA outcomes. Regression analyses revealed that prolonged SB (bouts >60 min) and total sedentary time were inversely associated with both baseline and peak BA diameters, with each additional hour of SB resulting in decreases of 0.08–0.1 mm in these diameters (*P* ≤ 0.01). Total sedentary time also showed similar negative associations with peak SFA diameters (β = −0.14 [−0.24 to −0.05], *P* < 0.01). Conversely, light-intensity PA and stepping time were positively associated with both baseline and peak BA diameters, with each additional hour increasing these diameters by 0.10–0.24 mm (*P* ≤ 0.02). Finally, standing time was positively associated with SFA peak diameter (β = 0.11 [0.01–0.20], *P* = 0.02). No associations were found between moderate-to-vigorous PA and vascular outcomes. In conclusion, in patients with RA, SB was negatively, whereas light PA was positively, associated with BA and SFA diameters. These findings suggest that reducing SB and increasing PA, even at light intensities, may improve vascular health in RA.

**NEW & NOTEWORTHY** This was the first study to investigate associations between objectively measured physical activity and markers of vascular health in rheumatoid arthritis (RA). The findings suggest that reducing sedentary behavior and increasing light or total physical activity are associated with improved vascular outcomes in RA. These results support further investigation into interventions aimed at reducing sedentary time and replacing with any type of physical activity as a potential strategy for improving cardiovascular outcomes in individuals with RA.

## Graphical abstract

**Figure:**
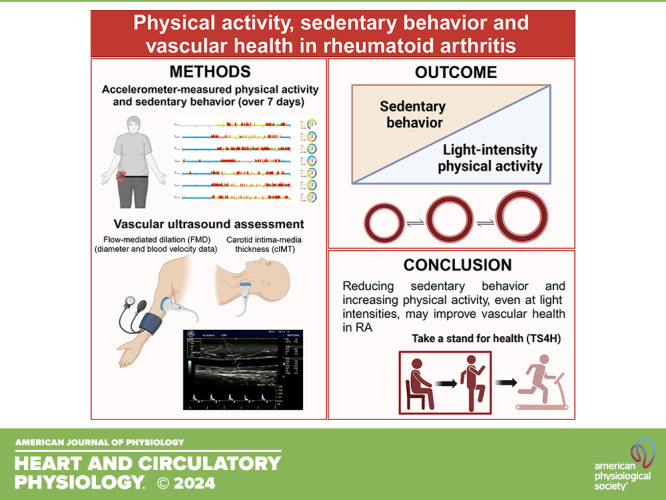

## INTRODUCTION

Rheumatoid arthritis (RA) is a chronic autoimmune inflammatory disorder characterized by accelerated atherosclerosis and increased cardiovascular risk (1, 2). Interventions aimed to reduce cardiovascular risk in RA should target the initial stages of the atherosclerosis continuum, which are characterized by endothelial dysfunction, subclinical atherosclerosis, and inward vascular remodeling (3).

Moderate-to-vigorous physical activity (MVPA) has been listed as an effective intervention to support the management of cardiovascular disease and to improve vascular health in the general population (4) and in patients with RA (5). However, individuals with RA usually present with low levels of MVPA (6), which may be due to disease-specific barriers for performing higher intensity physical activity, such as high levels of fatigue, and joint pain and stiffness (7).

More recent evidence suggests that in addition to performing MVPA, reducing sedentary time (e.g., sitting or lying down) and replacing it with lighter activities (e.g., standing or mild stepping) offers significant health benefits for individuals with RA (8, 9). For instance, a previous study from our group demonstrated that brief active breaks during prolonged sitting improve glycemic control and reduce inflammatory markers in women with RA (8). These benefits may also extend to the vasculature (10). However, potential associations between sedentary behavior/physical activity and markers of vascular function and structure in RA remain largely unexplored, warranting further research to inform the design of appropriate behavioral interventions.

To the best of our knowledge, only one previous study investigated the associations between sedentary time and vascular outcomes in RA, demonstrating that increased time spent in sitting position is associated with impaired endothelial function in the micro- but not in the macrovasculature (11). However, the previous study used self-reported sitting time to estimate sedentary behavior, which varies significantly between individuals, is less accurate than objective device-based methods (12), and lacks details on physical activity intensity, limiting the study’s findings. In addition, it measured vascular function in upper limb vessels by assessing forearm skin blood flow velocity in response to iontophoresis of acetylcholine and sodium nitroprusside, along with brachial artery flow-mediated dilation (FMD). However, evidence suggests that lower-limb arteries may benefit more from reducing sitting time due to restoration of leg blood flow, which enhances shear stress and endothelial function (13).

Therefore, our study aimed to expand previous findings and investigate the associations between objectively-measured sedentary behavior and physical activity levels and upper and lower limb endothelial function (i.e., FMD and associated measurements) and markers of subclinical atherosclerosis [i.e., carotid intima-media thickness (cIMT)] in postmenopausal women with RA. We hypothesized that increased sedentary behavior and reduced physical activity levels would associate with deteriorated endothelial function (i.e., reduced FMD), inward vascular remodeling (i.e., reduced arterial diameters), and accelerated atherosclerosis, whereas reduced sedentary behavior and higher levels of physical activity would associate with an improved vascular profile in postmenopausal women with RA.

## MATERIALS AND METHODS

### Study Design

This was a cross-sectional exploratory study nested within a randomized controlled trial (NCT03186924) (14, 15) conducted at the Laboratory of Assessment and Conditioning in Rheumatology (Clinical Hospital, University of Sao Paulo, Brazil). The participants included in this study represent a subsample of the trial and were recruited between July 2018 and February 2022. All data collected for the present study and reported herein refers to the preintervention assessment of the previous trial.

### Participant Recruitment and Ethical Approval

Postmenopausal women diagnosed with rheumatoid arthritis (16) were recruited from the Rheumatoid Arthritis Outpatient Clinic of the Clinical Hospital (University of Sao Paulo, Brazil). The exclusion criteria included: *1*) participation in structured exercise training programs within the past 12 mo; *2*) unstable drug therapy in the past 3 mo prior to and during the study; *3*) Health Assessment Questionnaire score >2.0; and *4*) presence of heart or renal disease, and stroke.

Prior to participation in the study, participants received a detailed explanation about the experimental procedures and provided their written consent. The study followed the principles of the Declaration of Helsinki and was approved by the local Institutional Ethics Committee.

### Assessments and Procedures

Assessments took place in two separated visits to the laboratory. During the first visit, clinical parameters were assessed, and participants were provided with accelerometers to monitor sedentary behavior and physical activity. The second visit was scheduled for the vascular assessment. Both visits were booked within a 2-wk interval.

#### Clinical parameters.

Disease activity was assessed by the Disease Activity Score-28 for Rheumatoid Arthritis with C reactive protein (DAS28-CRP) (17) and the Clinical Disease Activity Index (CDAI) (18). Pain levels were assessed through a 10-point Visual Analog Scale (VAS). The Health Assessment Questionnaire (HAQ) (19) was used to evaluate functional capacity in different domains of daily life. Additional clinical data (e.g., disease duration, comorbidities, and medication use) were obtained by the review of recent (<6 mo) medical records.

#### Sedentary behavior and physical activity levels.

Sedentary behavior (lying down and sitting), standing, and stepping time were measured using activPAL-micro (PAL Technology, Glasgow, UK). Patients wore the accelerometer on the right medial front thigh for seven consecutive days (24 h/day). The accelerometer data were imported into and analyzed using the PALanalysis software (v.7.2.32, PAL Technology, UK). The following data were reported: time spent in sedentary behavior (i.e., sitting and lying down, h/day), time spent in prolonged sitting in bouts >60 min (h/day), standing (h/day), and stepping (h/day), and number of breaks in sedentary time.

All data were standardized to a 16-h day to avoid bias from differences in participants’ daily wear time, using the formula: (data × 16)/wear time (14).

#### Physical activity level.

Physical activity levels were objectively measured using actiGraph GT3X (ActiGraph). All patients were instructed to wear the accelerometer during waking hours for seven consecutive days. The device was worn on a belt at the waistline on the right side of the hip. Data were exported in 60-s epochs using ActiLife 6 software (v.6.11.9, ActiGraph). Participants had to accumulate at least 10 h of valid activity recordings per day for at least 4 days, including one weekend day. Freedson cut-points were used to define time spent in each exercise intensity, as follows: light-intensity physical activity (LPA) (≥100 to <1,952 counts/min), and MVPA (≥1,952 counts/min) (20). Data were reported as follows: LPA (h/day) and MVPA (min/day).

All data were standardized to a 16-h day to avoid bias from differences in patients’ wear time, using the formula: (data × 16)/wear time (14).

#### Vascular ultrasound assessments.

Participants reported to the laboratory in the afternoon for the assessment of brachial (BA) and superficial femoral artery (SFA) flow-mediated dilation (FMD), and common carotid artery intima-media thickness (cIMT). These assessments were performed by an experienced sonographer using a high-resolution ultrasound machine (LOGIQ-e PRO, GE-Healthcare) equipped with a 4.0–12.0 MHz linear transducer. The experiments were conducted in the afternoon between 12:00 PM and 6:00 PM. Participants were instructed to abstain from eating food for a minimum of 4 h, smoking for a minimum of 6 h, avoid caffeine-containing beverages for at least 12 h, and intense exercise for 24 h prior to the tests, while maintaining their regular medication regimen. They were asked before the assessment whether they had followed these instructions and whether they had changed their medication regimen in the days prior. If participants did not adhere to the instructions or altered their medication routine, the test was rescheduled to a later date to ensure compliance with the protocol.

##### Brachial and superficial femoral artery flow-mediated dilation—FMD.

Assessments of FMD in BA and SFA were performed according to current guidelines (21). Testing was performed first in BA, with a 15-min interval between tests.

For BA, participants extended their right arm ∼80° from the torso and researchers immobilized it with foam supports. The ultrasound transducer was positioned on the distal third of the participant’s arm while a manual pneumatic cuff was positioned at the forearm (2–3 cm below the elbow) to provide the ischemic stimulus. For SFA, participants were positioned with their right thigh externally rotated. The ultrasound transducer was placed on the mid-thigh and the cuff was positioned 1–2 cm above the knee. Longitudinal images of BA and SFA diameters were taken using the B-mode ultrasound, and simultaneous pulse-waved Doppler blood flow velocity were obtained using a <60° insonation angle with the sample volume placed in the center of the artery and aligned with the blood flow. Initially, a 1-min baseline recording of diameter and blood flow velocity of each investigated artery were performed and then the cuffs were inflated to ∼200 mmHg for 5 min. After cuff release, data were recorded for 3 min for BA and 5 min for SFA.

Offline analyses of artery diameters and shear rates were performed using a semiautomatic edge detection and wall tracking software (Cardiovascular Suite, Quipu, Italy). Baseline diameter was defined as the average vessel diameter measured during the 1-min baseline recording. Peak diameter was defined as the largest diameter observed following cuff release. FMD% was calculated as the percentage change of the vessel diameter after cuff release in relation to baseline vessel diameter [FMD = [(Peak Diameter | Baseline Diameter) / Baseline Diameter] × 100]. Shear rate was calculated as eight times the mean blood velocity divided by the internal diameter (Shear rate = 8 × mean blood velocity/internal diameter) (21). The relevant shear rate stimulus for the FMD response was calculated as the area under the curve of the shear rate up to the peak diameter (SRAUC). SRAUC was reported separately and was also used to normalize the FMD response (i.e., FMD/SRAUC).

##### Common carotid artery intima-media thickness—cIMT.

The assessment of cIMT was performed according to current guidelines (22). For that, patients remained with the head rotated and the ultrasound transducer was positioned longitudinally to the right common carotid artery (i.e., longitudinal plane), 1–2 cm below the carotid bulb. Ultrasound parameters (e.g., gain, depth, and focal zone) were modified to optimize the appearance of the intima border along the vessel.

Once a clear image of the vessel was obtained using B-mode ultrasound, 20–30 s video recordings were made at three distinct angles [anterior, lateral, and posterior (22)] for subsequent analysis. Analysis of cIMT was performed using an edge detection and wall tracking software (Cardiovascular Suite, Quipu, Italy). cIMT was measured at the distal wall of the common carotid artery (from the lumen-intima interface to the media-adventitia interface) and calculated as the average thickness over a vessel segment >10 mm in each of the three recorded angles across the 20–30 s of recording. cIMT_mean_ was calculated as the average of the cIMT in the three measured angles. In addition, common carotid diameter was calculated as the distance between the media-adventitia interfaces of both the near and far walls of the common carotid artery obtained throughout the video recordings. Common carotid artery wall-to-lumen ratio was calculated as the cIMT_mean_ divided by the mean carotid diameter.

### Statistical Analysis

Linear regression models were used to determine the association between sedentary behavior/physical activity variables and vascular outcomes, while controlling for potential cofounders. First, a simple linear model was fitted with the exposure (sedentary behavior/physical activity variables) as the outcome predictor (*model 1*). Then, a multiple linear regression model was constructed, including body mass index (BMI), age, and disease activity (DAS28-CRP) as additional covariates (*model 2*). Multicollinearity was assessed using variance inflation factor (VIF), and collinearity was disregarded if VIF < 5. All analyses were conducted in R (v.4.3.2), running in RStudio (v.2023.12.0.369, Posit Software, Boston, MA), using “car” library (v.3.1-2). Statistical significance was set as *P* < 0.05. Data are presented as means ± standard deviation or as otherwise specified.

## RESULTS

Of the 103 individuals who participated in our previous randomized trial (14), 82 agreed to take part and were included in the analysis of the present study. Importantly, 28 of the participants were recruited during the COVID-19 pandemic. All participants were asked whether they had experienced symptoms or received a diagnosis of COVID-19, and all reported that they did not have the virus at the time of their participation in the study. Participants’ characteristics are reported in Table 1. Average sample age was 62 yr and participants were mostly overweight (i.e., 72% had a BMI ≥ 25.0 kg/m^2^). Disease activity ranged from remission to high activity (mean DAS28-CRP was 3.4 ± 3.0, CDAI was 10.8 ± 9.9).

**Table 1.** Participants’ characteristics

Variables	All Participants *n* (%)
*n*	82
Age, yr	62 ± 7.6
BMI, kg/m^2^	28.8 ± 5.4
Disease parameters	
DAS28-CRP, AU	3.4 ± 3.0
CDAI, AU	10.8 ± 9.9
HAQ, AU	1.1 ± 0.6
Disease duration, yr	20 ± 11.0
Comorbidities	
Hypertension	47 (57%)
Dyslipidemia	42 (51%)
Type 2 diabetes	17 (21%)
Cardiovascular diseases	10 (12%)
Hypothyroidism	17 (21%)
Osteoarthritis	25 (30%)
Osteopenia or osteoporosis	23 (28%)
Fibromyalgia	17 (21%)
Lung diseases	9 (11%)
Sjögren syndrome	7 (9%)
Systemic lupus erythematosus	3 (4%)
Medication
DMARDs	70 (85%)
Prednisone	61 (74%)
Biological agents	45 (55%)
Non-steroidal anti-inflammatory drugs	32 (39%)
Antihypertensive drugs	47 (57%)
Antidyslipidemic drugs	42 (51%)
Antidiabetic drugs	17 (21%)
Pain killers	54 (66%)
Muscle relaxants	29 (35%)
Sedentary behavior and physical activity level	
Total sedentary behavior, h/day	8.2 ± 2.0
Prolonged sedentary behavior (bouts >60 min), h/day	1.6 ± 1.8
Breaks in sedentary behavior, number/day	45.2 ± 14.5
Standing time, h/day	5.9 ± 1.7
LPA, h/day	6.1 ± 1.5
MVPA, min/day	18.0 ± 15.2
Stepping time, h/day	1.71 ± 0.67

Data are presented as means ± SD. AU, arbitrary units; BMI, body mass index; CDAI, Clinical Disease Activity Index; DAS28, Disease Activity Score in 28 joints; DMARDS, disease-modifying antirheumatic drugs; HAQ, Health Assessment Questionnaire; LPA, light intensity physical activity; MVPA, moderate-to-vigorous physical activity; PA, physical activity.

Sedentary behavior and physical activity levels are reported in Table 1. On average, participants spent a total of 8.2 h/day in sedentary behavior, of which 1.6 h/day were spent in prolonged (>60 min) bouts. Participants averaged 18 min/day in MVPA. Data from the assessments of vascular function and structure are reported in Table 2.

**Table 2.** Vascular parameters

Variables	All Participants
BA	
BA baseline diameter, mm	3.7 ± 0.5
BA peak diameter, mm	3.9 ± 0.5
BA-FMD, %	4.3 ± 3.0
BA-shear rate AUC, AU × 10^3^	38.7 ± 21.1
BA-FMD/ shear rate AUC, AU × 10^−5^	0.156 ± 0.197
SFA	
SFA baseline diameter, mm	5.3 ± 1.0
SFA peak diameter, mm	5.6 ± 0.8
SFA-FMD, %	3.7 ± 2.2
SFA-shear rate AUC, AU × 10^3^	22.1 ± 17.5
SFA-FMD/ shear rate AUC, AU × 10^−5^	0.303 ± 0.426
Common carotid artery	
Carotid diameter mean, mm	6.8 ± 0.7
cIMT_mean_, mm	0.7 ± 0.1
Wall-to-lumen ratio	0.108 ± 0.017

Data are presented as means ± SD. AU, arbitrary units; AUC, area under the curve; BA, brachial artery; cIMT_mean_, mean common carotid intima-media thickness; FMD, flow-mediated dilation; SFA, superficial femoral artery.

Results from the regression analyses examining the associations between objectively measured sedentary behavior and vascular outcomes are reported in Table 3. Prolonged sedentary behavior (in bouts >60 min) was inversely associated with both baseline and peak BA diameters. Each additional hour/day of prolonged sedentary behavior corresponded to reductions of 0.07 mm and 0.08 mm in baseline and peak BA diameters, respectively, in *model 1* (*P* = 0.03 for both). These associations persisted after adjusting for confounders in *model 2*, in which each additional hour/day of prolonged sedentary behavior was associated with a 0.1 mm decrease in both baseline and peak BA diameters (*P* < 0.01 for both). In the adjusted model, total sedentary time was also inversely associated with BA diameters, with each additional hour of sedentary behavior corresponding to reductions of 0.08 mm in baseline and peak BA diameters (*P* = 0.01 and *P* < 0.01, respectively). A similar relationship was observed for SFA peak diameter and total sedentary time, in which each additional hour of sedentary behavior was associated with a 0.14 mm reduction in peak SFA diameter (*P* < 0.01) in *model 2*. BA FMD/SRAUC was positively associated with number of breaks in sedentary time in *model 1*, however these associations were no longer present after adjusting for confounders (in *model 2*). There were no significant associations between sedentary behavior and any of the other FMD-related variables or any of the cIMT parameters across all models.

**Table 3.** Results of regression analyses examining associations between sedentary behavior and vascular function and structure

	Sedentary Behavior, h/day					Prolonged Sedentary Behavior, h/day					Breaks, number/Day				
	β	*P*	95% CI	*R* ^2^	*P* Model	β	*P*	95% CI	*R* ^2^	*P* Model	β	*P*	95% CI	*R* ^2^	*P* Model
*Model 1*															
BA baseline diameter, mm	−0.05	0.07	−0.11; 0.01	0.04	0.07	−0.07	**0.03**	−0.14; −0.01	0.06	**0.03**	0.00	0.73	−0.01; 0.01	0.00	0.73
BA peak diameter, mm	−0.05	0.12	−0.11; 0.01	0.03	0.12	−0.08	**0.03**	−0.14; −0.01	0.06	**0.03**	0.00	0.71	−0.01; 0.01	0.00	0.71
BA-FMD, %	0.20	0.24	−0.14; 0.53	0.02	0.24	−0.02	0.93	−0.40; 0.37	0.00	0.93	0.01	0.81	−0.04; 0.06	0.00	0.81
BA SR AUC, AU × 10^3^	0.66	0.58	−1.74; 3.01	0.00	0.58	1.97	0.15	−0.70; 4.64	0.03	0.15	−0.15	0.38	−0.50; 0.19	0.01	0.38
BA-FMD/SR AUC, AU × 10^−5^	0.79	0.49	−1.46; 0.03	0.01	0.49	−1.26	0.33	−3.78; 1.27	0.01	0.33	0.31	**0.05**	0.00; 0.63	0.05	**0.05**
SFA baseline diameter, mm	−0.05	0.41	−0.16; 0.06	0.01	0.41	−0.02	0.75	−0.14; 0.10	0.00	0.75	−0.01	0.47	−0.02; 0.01	0.01	0.47
SFA peak diameter, mm	−0.09	**0.05**	−0.18; 0.00	0.05	**0.05**	−0.06	0.24	−0.16; 0.04	0.02	0.24	−0.01	0.27	−0.02; 0.01	0.02	0.27
SFA-FMD, %	0.12	0.35	−0.13; 0.36	0.01	0.35	0.02	0.87	−0.26; 0.30	0.00	0.87	0.03	0.12	−0.01; 0.06	0.03	0.12
SFA SR AUC, AU × 10^3^	0.23	0.82	−1.81; 2.28	0.00	0.82	0.46	0.69	−1.80; 2.73	0.00	0.69	0.03	0.99	−0.30; 0.30	0.00	0.99
SFA-FMD/SR AUC, AU × 10^−5^	0.60	0.81	−5.60; 4.42	0.00	0.81	1.69	0.55	−7.22; 3.84	0.00	0.55	−0.17	0.64	−0.93; 0.58	0.00	0.64
Mean common carotid diameter, mm	0.06	0.16	−0.02; 0.14	0.02	0.16	0.04	0.38	−0.05; 0.14	0.01	0.38	0.00	0.64	−0.02; 0.01	0.00	0.64
cIMT_mean_, mm	0.01	0.40	−0.01; 0.02	0.01	0.40	0.01	0.11	0.00; 0.02	0.03	0.11	0.00	0.90	0.00; 0.00	0.00	0.90
Common carotid wall-to-lumen ratio	0.00	0.55	0.00; 0.00	0.00	0.55	0.00	0.42	0.00;0.00	0.01	0.42	0.00	0.73	0.00; 0.00	0.00	0.73
*Model 2*															
BA baseline diameter, mm	−0.08	**<0.01**	−0.14; −0.02	0.16	**<0.01**	−0.10	**<0.01**	−0.16; −0.03	0.17	**<0.01**	0.00	0.48	−0.01; 0.01	0.08	0.17
BA peak diameter, mm	−0.08	**0.01**	−0.14; −0.02	0.12	**<0.01**	−0.10	**<0.01**	−0.17; −0.03	0.18	**<0.01**	0.00	0.44	−0.01; 0.01	0.09	0.13
BA-FMD, %	0.17	0.36	−0.19; 0.52	0.04	0.58	0.05	0.81	−0.36; 0.46	0.03	0.71	0.01	0.75	−0.04; 0.06	0.03	0.71
BA SR AUC, AU × 10^3^	0.66	0.61	−1.94; 3.26	0.01	0.96	1.84	0.21	−1.10; 4.74	0.03	0.74	−0.15	0.42	−0.51; 0.21	0.01	0.91
BA-FMD/SR AUC, AU × 10^−5^	1.14	0.34	−1.24; 3.52	0.05	0.40	−0.59	0.66	−3.28; 2.10	0.04	0.51	0.29	0.08	−0.03; 0.62	0.08	0.18
SFA baseline diameter, mm	−0.09	0.14	−0.21; 0.03	0.08	0.20	−0.02	0.72	−0.16; 0.11	0.05	0.42	0.00	0.65	−0.02; 0.00	0.05	0.41
SFA peak diameter, mm	−0.14	**<0.01**	−0.24; −0.05	0.18	**<0.01**	−0.07	0.21	−0.18; 0.04	0.09	0.13	−0.01	0.42	−0.02; 0.01	0.08	0.19
SFA-FMD, %	0.18	0.19	−0.10; 0.45	0.04	0.58	0.05	0.77	−0.25; 0.34	0.04	0.87	0.03	0.16	−0.01; 0.06	0.04	0.54
SFA SR AUC, AU × 10^3^	0.60	0.58	−1.56; 2.75	0.05	0.45	0.60	0.58	−1.56; 2.57	0.05	0.45	0.00	0.78	−0.04; 0.03	0.05	0.48
SFA-FMD/SR AUC, AU × 10^−5^	−1.25	0.64	−6.56; 4.05	0.05	0.48	−1.39	0.63	−7.22; 4.43	0.05	0.48	0.11	0.78	−0.88; 0.66	0.04	0.50
Mean common carotid diameter, mm	0.06	0.11	−0.01; 0.15	0.13	**0.03**	0.03	0.57	−0.06;0.12	0.10	0.09	0.00	0.95	−0.01; 0.01	0.10	0.10
cIMT_mean_, mm	0.00	0.37	−0.01; 0.02	0.15	**0.02**	0.01	0.45	−0.01; 0.02	0.15	0.02	0.00	0.58	0.00; 0.00	0.15	**0.02**
Common carotid wall-to-lumen ratio	0.00	0.96	0.00; 0.00	0.00	0.43	0.00	0.65	0.00; 0.00	0.00	0.40	0.00	0.74	0.00; 0.00	0.00	0.42

*Model 1*: simple linear regression. *Model 2*: adjusted by age, body mass index (BMI), and disease activity. *P* values in bold indicate statistical significance. AU, arbitrary units; AUC, area under curve until the peak dilation; BA, brachial artery; cIMTmean, mean common carotid intima-media thickness; FMD, flow-mediated dilation; SFA, superficial femoral artery; SR, shear rate.

No associations were observed between physical activity and any vascular parameters in *model 1*, as shown in Table 4. After adjusting for confounders, SFA peak diameter was positively associated with standing time, with each additional hour of standing corresponding to a 0.11 mm increase in peak SFA diameter (*P* = 0.02). BA diameters were positively associated with LPA, with each additional hour of LPA corresponding to increases in 0.1 mm in both baseline and peak diameters (*P* = 0.02). Finally, BA diameters were also positively associated with stepping time, with each hour increase in this activity corresponding to increases of 0.24 and 0.17 mm in baseline and peak diameter, respectively (*P* ≤ 0.01). There were no significant associations between physical activity and either FMD or any of the cIMT parameters across all models (Table 4).

**Table 4.** Results of regression analyses examining associations between physical activity levels and function and vascular structure

	Standing, h/day					Light-Intensity PA, h/day					Moderate-to-Vigorous PA, min/day					Stepping Time, h/day				
	β	*P*	95% CI	*R* ^2^	*P* Model	β	*P*	95% CI	*R* ^2^	*P* Model	β	*P*	95% CI	*R* ^2^	*P* Model	*β*	*P*	95% CI	*R* ^2^	*P* Model
*Model 1*																				
BA baseline diameter, mm	0.03	0.36	−0.03; 0.08	0.01	0.36	0.07	0.10	−0.01; 0.15	0.04	0.10	0.00	0.37	−0.01; 0.00	0.01	0.37	0.13	0.14	−0.04; 0.30	0.03	0.14
BA peak diameter, mm	0.02	0.46	−0.04; 0.08	0.01	0.46	0.07	0.11	−0.02; 0.15	0.03	0.11	0.00	0.41	−0.01; 0.00	0.01	0.41	0.12	0.20	−0.06; 0.29	0.02	0.20
BA-FMD, %	−0.15	0.35	−0.48; 0.17	0.01	0.35	−0.11	0.65	−0.57; 0.35	0.00	0.65	0.01	0.76	−0.04; 0.05	0.00	0.76	−0.50	0.32	−1.49; 0.49	0.01	0.32
BA SR AUC, AU × 10^3^	1.03	0.38	−3.38; 1.31	0.01	0.38	−1.51	0.36	−4.77; 1.75	0.01	0.36	0.11	0.51	−0.22; 0.44	0.01	0.51	−3.99	0.26	−11.07; 3.10	0.02	0.26
BA-FMD/SR AUC, AU × 10^−5^	0.79	0.48	−2.98; 1.42	0.01	0.48	0.14	0.93	−2.97; 3.24	0.00	0.93	0.15	0.36	−0.17; 0.46	0.01	0.36	1.10	0.74	−5.60; 7.80	0.00	0.74
SFA baseline diameter, mm	0.03	0.62	−0.08; 0.14	0.00	0.62	0.03	0.71	−0.12; 0.18	0.00	0.71	−0.01	0.25	−0.02; 0.01	0.02	0.25	−0.14	0.39	−0.47; 0.19	0.01	0.39
SFA peak diameter, mm	0.07	0.11	−0.02; 0.16	0.03	0.11	0.07	0.26	−0.05; 0.20	0.02	0.26	0.00	0.49	−0.02; 0.01	0.01	0.49	0.14	0.30	−0.13; 0.42	0.01	0.30
SFA-FMD, %	−0.05	0.66	−0.30; 0.19	0.00	0.66	0.04	0.83	−0.31; 0.38	0.00	0.83	0.00	0.80	−0.04; 0.03	0.00	0.80	−0.34	0.36	−1.07; 0.39	0.01	0.36
SFA SR AUC, AU × 10^3^	0.04	0.97	−1.98; 2.06	0.00	0.97	−0.64	0.65	−3.46; 2.18	0.00	0.65	−0.01	0.95	−0.03; 0.03	0.00	0.95	0.96	0.76	−5.18; 7.09	0.00	0.76
SFA-FMD/SR AUC, AU × 10^−5^	2.78	0.26	−2.12; 7.69	0.02	0.26	0.68	0.84	−6.27; 7.63	0.00	0.84	−0.23	0.51	−0.92; 0.00	0.01	0.17	−0.10	0.17	0.00; 4.43	0.03	0.17
Mean common carotid diameter, mm	−0.06	0.18	−0.14; 0.03	0.02	0.18	−0.10	0.08	−0.22; 0.01	0.04	0.08	0.00	0.50	−0.02; 0.01	0.01	0.50	−0.02	0.86	−0.27; 023	0.00	0.86
cIMT_mean_, mm	0.00	0.87	−0.01; 0.01	0.00	0.87	−0.01	0.40	−0.02; 0.01	0.01	0.40	0.00	0.12	0.00; 0.00	0.03	0.12	−0.02	0.21	−0.06; 0.01	0.02	0.21
Common carotid wall-to-lumen ratio	0.00	0.22	0.00; 0.00	0.02	0.22	0.00	0.47	0.00; 0.00	0.01	0.47	0.00	0.51	0.00; 0.00	0.01	0.51	0.00	0.41	−0.01; 0.00	0.01	0.41
*Model 2*																				
BA baseline diameter, mm	0.04	0.15	−0.02; 0.10	0.10	0.09	0.10	**0.02**	0.01; 0.18	0.13	**0.04**	0.00	0.81	−0.01; 0.01	0.06	0.34	0.24	**<0.01**	0.06; 0.42	0.16	**0.01**
BA peak diameter, mm	0.04	0.18	−0.02; 0.10	0.10	0.08	0.10	**0.02**	0.01; 0.18	0.13	**0.04**	0.00	0.87	−0.01; 0.01	0.06	0.30	0.17	**0.01**	0.05; 0.42	0.16	**0.01**
BA-FMD, %	−0.12	0.50	−0.47; 0.23	0.03	0.64	−0.12	0.63	−0.62; 0.38	0.02	0.84	0.01	0.70	−0.04; 0.06	0.02	0.85	−0.48	0.38	−1.56; 0.60	0.04	0.58
BA SR AUC, AU × 10^3^	−1.21	0.33	−3.71; 1.27	0.02	0.86	−1.50	0.40	−5.04; 2.05	0.02	0.83	0.16	0.38	−0.20; 0.52	0.02	0.82	−4.28	0.27	−12.00; 3.45	0.02	0.81
BA-FMD/SR AUC, AU × 10^−5^	−0.72	0.53	−3.02; 1.58	0.05	0.48	−0.43	0.80	−3.75; 2.88	0.05	0.49	0.09	0.61	−3.75; 2.88	0.05	0.46	−1.20	0.97	−7.29; 7.04	0.04	0.54
SFA baseline diameter, mm	0.06	0.35	−0.06; 0.17	0.06	0.32	0.05	0.50	−0.11; 0.22	0.04	0.59	−0.01	0.36	−0.02; 0.01	0.04	0.52	−0.08	0.64	−0.44; 0.27	0.05	0.41
SFA peak diameter, mm	0.11	**0.02**	0.01; 0.20	0.14	**0.03**	0.10	0.12	−0.03; 0.23	0.08	0.20	0.00	0.68	−0.02; 0.01	0.06	0.43	0.24	0.10	−0.05; 0.53	0.10	0.08
SFA-FMD, %	−0.09	0.50	−0.35; 0.17	0.02	0.81	−0.02	0.91	−0.39; 0.35	0.01	0.94	0.01	0.79	−0.04; 0.03	0.01	0.93	−0.50	0.22	−1.30; 0.31	0.04	0.61
SFA SR AUC, AU × 10^3^	0.28	0.79	−2.37; 1.81	0.05	0.48	−0.83	0.58	−3.79; 2.13	0.04	0.58	0.03	0.85	−0.33; 0.27	0.03	0.63	0.52	0.87	−5.89; 6.93	0.05	0.49
SFA-FMD/SR AUC, AU × 10^−5^	3.69	0.15	−1.38; 8.75	0.07	0.25	1.15	0.75	−6.15; 8.45	0.05	0.51	0.25	0.51	−0.98; 0.49	0.05	0.46	−0.10	0.17	−0.26; 4.83	0.07	0.27
Mean common carotid diameter, mm	−0.07	0.09	−0.14; 0.01	0.14	**0.03**	−0.11	0.05	−0.21; 0.01	0.15	**0.02**	0.00	0.91	−0.01; 0.01	0.11	0.08	−0.03	0.79	−0.28; 0.21	0.10	0.09
cIMT_mean_, mm	0.00	0.67	−0.01; 0.01	0.15	**0.02**	−0.01	0.33	−0.02; 0.01	0.16	**0.02**	0.00	0.38	0.00; 0.00	0.15	**0.02**	−0.02	0.23	−0.06; 0.01	0.16	**0.01**
Common carotid wall-to-lumen ratio	0.00	0.39	0.00; 0.00	0.05	0.46	0.00	0.71	0.00; 0.00	0.06	0.40	0.00	0.43	0.00; 0.00	0.06	0.33	0.00	0.33	−0.01; 0.00	0.05	0.43

*Model 1*: simple linear regression. *Model 2*: adjusted by age, body mass index (BMI) and disease activity. *P* values in bold indicate statistical significance. AU, arbitrary units; AUC, area under the curve until the peak dilation; BA, brachial artery; cIMTmean, mean common carotid intima-media thickness; FMD, flow-mediated dilation; SFA, superficial femoral artery; SR, shear rate.

## DISCUSSION

The present study tested the associations between objectively measured sedentary behavior and physical activity and markers of macrovascular endothelial function and carotid artery structure in RA. Our results indicated that sedentary behaviors were inversely associated, whereas light forms of physical activity (LPA and standing) and total physical activity (stepping time) were directly correlated with BA and SFA diameters, both at rest and after reactive hyperemia. These associations were independent of BMI, disease severity, and age. On the other hand, there were no significant associations between physical activity or sedentary behavior with FMD or any of the cIMT parameters.

Lack of associations between FMD in the BA and SFA and physical activity and sedentary behavior contradicts the study hypothesis and previous studies showing positive associations between physical activity and FMD (5, 23). However, this finding should be interpreted in light of the observed associations between physical activity, sedentary behavior, and the diameters of the BA and SFA. Previous studies have reported complementary and reciprocal changes in FMD and arterial diameters in response to physical activity (24–26). These studies suggest that FMD and arterial diameters adapt to physical activity according to two distinct time courses, with initial improvements in FMD being replaced over time by increase in artery diameters (i.e., arterial remodeling) and return of FMD toward baseline levels. Due to the cross-sectional nature of this study, it is not possible to capture these time-related associations between physical activity data and FMD and arterial diameter. However, based on our results, it is possible to speculate that the participants with lower sedentary behavior and higher physical activity levels had already gone through this adaptive process, reaching the point of increased arterial diameters and return of FMD to baseline levels. This would explain the observed associations between physical activity data and BA and SFA diameters, alongside the absence of associations with FMD. This hypothesis should be tested in longitudinal studies exploring the temporal relationship between physical activity, FMD, and arterial remodeling in RA.

The observed associations between increased physical activity levels and/or reduced sedentary behavior with increased BA and SFA diameters, nevertheless, suggest that various PA behaviors (e.g., reducing prolonged or total sedentary time or increasing light intensity PA or stepping time) may positively affect vascular phenotype in RA. Importantly, RA is associated with an elevated cardiovascular risk (1, 2), partly due to an altered vascular profile caused by both (dys)functional changes [e.g., endothelial dysfunction (1), vessel inflammation (27), and sympathetic hyperactivity (28)] and structural inward vascular remodeling (29). Indeed, the average FMD in our study sample was 4.3% for the brachial artery, which is below the age-adjusted 50th percentile for brachial FMD in females (30) and aligns with a classification of impaired endothelial function (31), confirming the presence of endothelial dysfunction in the study sample. These vascular alterations represent key hallmarks of the early stages of the cardiovascular disease continuum, which may culminate with the development of advanced cardiovascular disease. Therefore, the results of the present study suggest a potential counteracting role of physical activity on some of these adverse vascular changes, which may contribute to reduce cardiovascular risk in RA.

The results of the present study indicate that reduced sedentary behavior and increased light physical activity are associated with improved vascular markers in both upper- and lower-limb arteries. Previous studies that modulated sedentary time through prolonged sitting and/or via active breaks in these prolonged sitting have suggested that lower limb arteries may be more responsive than upper limb arteries to changes in sedentary time and physical activity (10, 13). However, this trend was not confirmed by our regression analyses. The observational design and tools used in the present study do not allow us to draw definitive conclusions about the absence of these expected limb-specific associations between vascular parameters with sedentary behavior and physical activity. However, inconsistencies between our findings and those of previous studies may reflect the different types of sedentary behaviors captured in our study, which may include not only sitting but also reclining and lying down positions. Sitting is more likely to reduce shear rate and affect the function of lower limb arteries (10, 13, 32), while reclining and lying down may not have the same impact on these arteries.

The lack of significant associations between physical activity or sedentary behavior and cIMT—a marker of subclinical atherosclerosis—is inconsistent with the study hypothesis. However, this indicates that physical activity alone does not appear to be associated with an improved atherosclerotic profile in RA. This finding aligns with the notion that a combination of multiple factors (e.g., physical activity, smoking cessation, dietary changes, and lipid-lowering drugs) (33) may be required to reverse atherosclerosis in clinical populations, an hypothesis that needs to be tested by studies using multicomponent interventions. Alternatively, longer-duration physical activity behavior (>6 mo) may also be associated with an improved atherosclerotic profile (34), which should be tested in longitudinal studies.

Interestingly, although our study found associations between lighter forms of physical activity and total physical activity with some vascular outcomes, no association was observed between MVPA and any vascular outcome. Although it’s widely understood that all forms of physical activity positively impact health, evidence also suggests a dose-response relationship, with higher intensities yielding the greatest benefits (35), a finding not supported by the present study on postmenopausal women with RA. These results, however, need to be interpreted considering that average daily time spent in MVPA was low, with only ∼25% of subjects meeting recommendations from guidelines (i.e., 150 min/wk of MVPA), a fact that may have blunted potential associations. Notwithstanding, device-derived MVPA is associated with all-cause mortality risk reduction even at lower levels of activity (35), and increases of any magnitude in this behavior should be encouraged in this population.

Taken together, our findings emphasize the potential benefits of promoting physical activity of any intensity in patients with RA. The observed associations between reducing sedentary time and increasing lighter forms of PA with improved vascular diameters are encouraging, as some patients with RA may find it challenging to perform MVPA due to recurrent pain and fatigue (7). In contrast, engagement in light physical activity may be more easily achievable by these patients and may possibly modulate cardiovascular risk in people with RA. In addition, engagement in light physical activity may, over time, increase patients’ confidence and physical capacity to participate in moderate or vigorous activities. In this way, light activity may serve as a gateway to more intense physical activity. These notions align with current exercise guidelines for RA, which emphasize the importance of tailoring exercise programs to each patient’s capabilities (36).

Our study does not come without limitations, including its cross-sectional approach, inherently hindering causal inferences. The study sample included only postmenopausal women with RA, so the findings cannot be directly generalized to other populations, such as men or women of reproductive age. Specifically related to the FMD assessments, the study used a manual pneumatic cuff, which arguably results in slower cuff inflation/deflation compared with automatic systems, potentially affecting the shear stress response and the resulting dilation. The study results should also be interpreted considering the potential effects of cardiovascular, antihypertensive, and lipid-lowering medications, which may have influenced vascular outcomes and modulated the associations between physical activity and vascular function in our study sample. In addition, caution should be exercised when interpreting the study findings, considering that physical activity/sedentary behavior showed no associations with more broadly used measures of vascular health (i.e., FMD and cIMT). Furthermore, the low levels of MVPA observed in the sample might have affected possible associations between this behavior and vascular outcomes, as previously discussed. Nonetheless, our study is the first, to the best of our knowledge, to explore and demonstrate existing associations between objectively measured sedentary and physical activity with vascular outcomes in RA, advancing the current knowledge with more reliable and accurate measures of physical activity and sedentary behavior.

In conclusion, reduced sedentary behavior and increased light-intensity and total physical activity are associated with improved vascular outcomes in RA. Our results suggest the potential role of replacing sedentary behavior with light physical activity to improve vascular health markers in RA. However, as this was a cross-sectional study, these findings need to be tested in specific intervention trials before they can be recommended as a standard strategy for managing vascular health in patients with RA.

## DATA AVAILABILITY

Data will be made available upon reasonable request.

## GRANTS

Kamila Meireles dos Santos was supported by a grant from the Coordenação de Aperfeiçoamento de Pessoal de Nível Superior (CAPES). Ana Jessica Pinto, Tiago Peçanha, Lucas Porto Santos, and Bruno Gualano were supported by the Fundação de Amparo à Pesquisa do Estado de São Paulo (FAPESP) under Grant Nos. 2015/26937-4 and 2018/19418-9; 2016/23319-0 and 2019/07150-4; 2022/12890-0; 2017/13552-2. Tiago Peçanha and Hamilton Roschel were supported by Conselho Nacional de Desenvolvimento Científico e Tecnológico (CNPq) under Grant Nos. 406196/2018-4; 428242/2018-9. Claudia L. M. Forjaz was supported by Conselho Nacional de Desenvolvimento Científico e Tecnológico (CNPq) Grant 302309/2022-5.

## DISCLOSURES

No conflicts of interest, financial or otherwise, are declared by the authors.

## AUTHOR CONTRIBUTIONS

K.M., T.P., A.J.P., A.C.M.R., A.L.d.S.P., F.R.L., B.G., and H.R. conceived and designed research; K.M., T.P., A.J.P., L.P.S., B.C.M., F.I.S., D.R., and N.D.d.S.J. performed experiments; K.M., T.P., A.J.P., L.P.S., and B.C.M. analyzed data; K.M., T.P., A.J.P., L.P.S., B.C.M., F.I.S., C.L.M.F., B.G., and H.R. interpreted results of experiments; K.M., T.P., A.J.P., and L.P.S. prepared figures; T.P., A.J.P., L.P.S., C.L.M.F., and H.R. drafted manuscript; K.M., T.P., A.J.P., L.P.S., B.C.M., F.I.S., D.R., A.C.M.R., A.L.d.S.P., F.R.L., N.D.d.S.J., C.L.M.F., B.G., and H.R. edited and revised manuscript; K.M., T.P., A.J.P., L.P.S., B.C.M., F.I.S., D.R., A.C.M.R., A.L.d.S.P., F.R.L., N.D.d.S.J., C.L.M.F., B.G., and H.R. approved final version of manuscript.
